# Introduction of vasculature in engineered three-dimensional tissue

**DOI:** 10.1186/s41232-017-0055-4

**Published:** 2017-12-01

**Authors:** Sachiko Sekiya, Tatsuya Shimizu

**Affiliations:** Institute of Advanced Biomedical Engineering and Science, Tokyo Women’s Medical University, 8-1 Kawada-cho, Shinjuku-ku, Tokyo, 162-8666 Japan

**Keywords:** Induction vascularization, Selecting cells and material, Tissue engineering, Assemble, Perfusion, Three-dimensional tissues

## Abstract

**Background:**

With recent developments in tissue engineering technology, various three-dimensional tissues can be generated now. However, as the tissue thickness increases due to three-dimensionalization, it is difficult to increase the tissue scale without introduction of blood vessels.

**Main text:**

Many methods for vasculature induction have been reported recently. In this review, we introduced several methods which are adjustable vascularization in three-dimensional tissues according to three steps. First, “selection” provides potents for engineered tissues with vascularization ability. Second, “assembly technology” is used to fabricate tissues as three-dimensional structures and simultaneously inner neo-vasculature. Third, a “perfusion” technique is used for maturation of blood vessels in three-dimensional tissues. In “selection”, selection of cells and materials gives the ability to promote angiogenesis in three-dimensional tissues. During the cell assembly step, cell sheet engineering, nanofilm coating technology, and three-dimensional printing technology could be used to produce vascularized three-dimensional tissues. Perfusion techniques to perfuse blood or cell culture medium throughout three-dimensional tissues with a unified inlet and outlet could induce functional blood vessels within retransplantable three-dimensional tissues. Combination of each step technology allows simulation of perivascular microenvironments in target tissues and drive vascularization in three-dimensional tissues.

**Conclusion:**

The biomimetic microenvironment of target tissues will induce adequate cell-cell interaction, distance, cell morphology, and function within tissues. It could be accelerated for vascularization within three-dimensional tissues and give us the functional tissues. Since vascularized three-dimensional tissues are highly functional, they are expected to contribute to the development of regenerative medicine and drug safety tests for drug discovery in the future.

## Background

Tissue engineering (TE) technologies have been progressing recently. The development of these technologies has produced dramatic effects on cell transplantation therapy [1, 2]. Moreover, three-dimensional (3D) structures fabricated from cells express important functions and the differentiation capacity of stem cells in vitro. These 3D tissues will be also available as tools for safety tests on chemical substances or for drug discovery. Indeed, a reduction in the use of animals for laboratory experiments is required globally for the drug development process and other applications, from the perspective of animal welfare. The use of animal-free technology to fabricate tissues will accelerate this reduction.

**Fig. 1:**
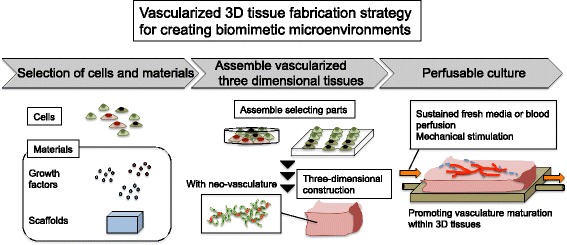
Vascularized 3D tissue fabrication strategy for creating biomimetic microenvironments. The figure shows a flow chart of vessel induction strategies according to three steps: selecting cells and materials for vascularized ability within 3D tissues, assembly technology as the method of 3D fabrication which control distribution and promotion of vascularization, and perfusable culture for functional vascular maturation

As the thickness of engineered 3D tissue increases, however, induction of inner vasculature is required in order to supply oxygen and nutrients, including fatty acids, and remove waste products. In typical two-dimensional (2D) cell culture conditions, the thickness of the cell population is approximately 20–30 μm, which is sufficient to allow diffusion of nutrients and oxygen. When the thickness of engineered tissues exceeds 100 μm, the oxygen and nutrients are difficult to diffuse to the inner side of the tissue [3]. Therefore, to resolve this thickness issue, introducing blood vessels into 3D engineered tissues has been studied, and various methodologies to achieve this have been established [4, 5]. For example, tissues exceeding 1 mm in thickness can be obtained in vivo when the 80-μm layered cell sheets are multistep-transplanted with a vascular linkage between each layered cell sheet [6]. Moreover, a perfusable system and micro-perfusable channel have recently been developed in vitro for 3D tissue vascularization. In this review, we will introduce the latest vessel induction strategies according to three steps: selecting cells and materials for vascularized 3D tissues, assembly selecting parts as vascularized 3D shapes, and promotion of vascularization, with perfusable culture (Fig. 1). Combination of these technologies will produce physiological mimic microenvironment in vivo and could drive vascularization for target engineered tissues. Such biomimetic microenvironments can approach the engineered tissues close to the ideal function and structure.

### Selecting cells and materials for vascularization into 3D engineered tissues

**Fig. 2:**
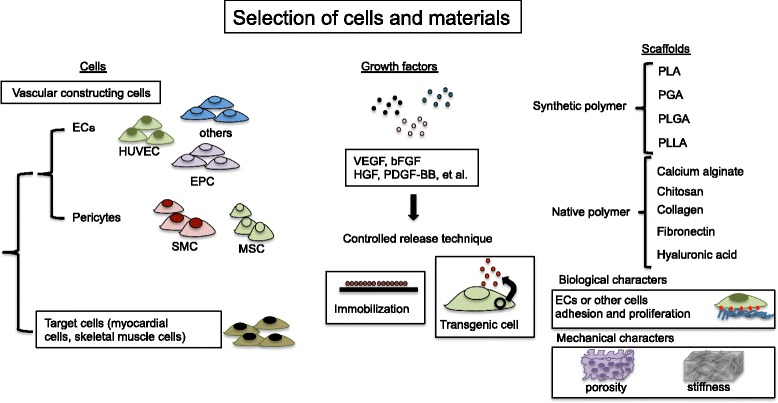
Selection of cells and materials. The figure shows several candidates of potent cells and materials including activating growth factor and promoting scaffold within 3D tissues for conditioning vascularization microenvironments

During fabrication of 3D tissues from cells, it is necessary to induce the generation of blood vessels simultaneously. For conditioning vascularization microenvironments, we have to choose potent cells and materials including activating growth factor and promoting scaffold within 3D tissues (Fig. 2).

Cells constructing vasculature, endothelium-constructed endothelial cells (human umbilical vein endothelial cell: HUVEC, endothelial progenitor cell: EPC, and other kinds of endothelial cell: EC), and perivascular-constructed cells (mesenchymal stem cell: MSC and smooth muscle cell: SMC) could be considered as potent cells for vascularization within tissues. Selecting these cells is dependent on kinds of target tissue.

Simply coculturing cells is a technique to induce blood vessels within engineering of 3D tissues. Secreting cytokines and other factors, including cell adhesion factors and extracellular matrix (ECM), from cocultured cells induce the neo-vasculature within 3D tissues. Previously, myocardial sheets with a vascular EC network structure could be fabricated by cultivation with vascular ECs and fetal left ventricle-derived cardiomyocytes [7]. The myocardial cell sheet contained not only ECs and myocardial cells but also fibroblasts and pericytes. This EC network containing myocardial tissue was able to promote blood circulation shortly, which guarantees the survival and growth of 3D tissues after transplantation in vivo [8]. Actually, the tricultured scaffold with ECs, myoblasts, and fibroblasts also induced vasculature within 3D tissues in vitro [9]. In contrast, the EC network can also observe during differentiation into hepatocytes from endoderm-differentiated induced pluripotent stem (iPS) cells by coculture [10] and renal tubular cells from iPS cells [11]. These EC networks within primitive tissues are probably similar to the primary vascular plexus during the embryonic period, which is associated with the supply of blood flow promptly into immature tissues during development. Thus, EC networks are considered as one better indicator of selecting cells for vascularized 3D tissues.

The EC network structure could be also induced by coculture with dermal fibroblasts, skeletal myoblasts, adipose-derived MSCs (ADMSCs), and bone marrow-derived MSCs (BMMSCs) [12–14]. In particular, MSCs could differentiate into vascular ECs [15], and pericytes could be also considered as adipose-derived MSCs [16]. Thus, MSCs have probably potents for promotion angiogenesis within the engineered 3D tissue. Notably, after transplantation, MSC-containing 3D tissues showed greater regeneration than that without MSCs by inducing macrophage infiltration [17]. Macrophage infiltration due to inflammation alters the EC network structure in vitro and promotes angiogenesis in vivo [18]. MSCs also exhibit immunomodulation after bone marrow transplantation [19]. This ability of MSCs to affect inflammation may accelerate induction of vascularized 3D tissues in vivo.

Growth factor is also important for vascularization of 3D tissues. Culture medium containing growth factor is well known to induce vascularization in 3D tissues [20]. However, angiogenesis-promoting factors, vascular endothelial growth factor (VEGF), basic fibroblast growth factor (bFGF), hepatocyte growth factor (HGF), platelet-derived growth factor-BB (PDGF-BB), and angiopoietin-1 have common issues, quickly degradation and diffusion. To overcome these issues, we immobilized them with scaffolds [21] or co-cultured with VEGF transgenic cells [22]. It is also able to administrate sustained growth factors for local interested sites and cause gradients of growth factors [23]. It was reported that microvasculature is induced at the transplant position before transplantation via administration of a sustained-release VEGF or bFGF, to enhance vascularization of implanted 3D tissues [24]. Thus, the controlled release growth factors can be useful for vascularization within engineered 3D tissues.

Selecting scaffold materials (e.g., synthetic polymers and natural polymers) is also important for vascularization of 3D tissues [25]. Co-polymer of poly lactic acid (PLA) and poly glycolic acid (PGA) and poly-(L-lactide) (PLLA) and poly –(lactic-co-glycolic) acid (PLGA) are well known as synthetic biodegradable polymers for 3D tissue fabrication. Natural polymers, collagen, fibronectin, and hyaluronic acid are also well utilized for vascularization of 3D tissues. Especially, extracellular matrix component could affect ECs adhesion and proliferation. These polymers could combine with each other for 3D tissue fabrication. In prior studies, well-vascularized 3D skeletal muscle tissues were fabricated in vivo with PLLA/PLGA scaffolds [26]. The EC network structures have been obtained within tri-cell cultured 3D tissues by adding fibrin to PLLA/PLGA scaffold during cultivation [27]. Moreover, mechanical characters (e.g., porous size and stiffness) of scaffold affected for vascularization ability within 3D tissues. The vascularization within the 3D tissue is probably controlled scaffold size, mechanical or chemical character optimization of the copolymer biodegradation time [28]. Good selecting materials as scaffolds will mediate for vascularized 3D tissues.

Summarizing, suitable selective cells and materials are an important step for vascularization ability, EC-network formation, and vascular density inner 3D engineered tissues.

### Assembly of cells into vascularized 3D engineered tissues: cell manipulation and scaffold shaping

**Fig. 3:**
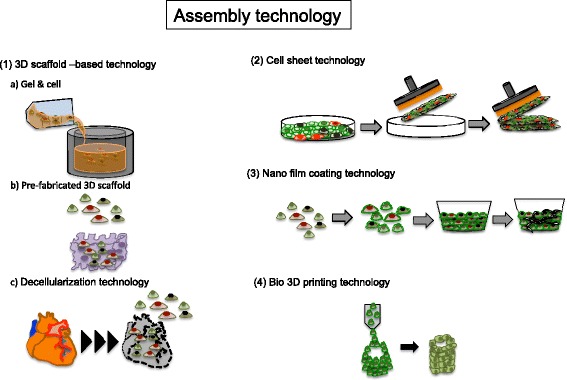
Assembly technology. The figure shows representative assembly technology for fabrication of 3D tissues with vasculature

Following selection step, cells and materials have to be arranged artificially or efficiently self-organization. Therefore, the assembly of cells and materials is another key point for fabrication of 3D tissues with vasculature (Fig. 3).

Recently, the thin coating of proteins on individual cell surfaces has been reported to facilitate the fabrication of 3D tissues without a scaffold in vitro [29]. The coated cells are cultured on a porous permeable membrane. After adhesion of the basic layer, the next layer of cells is cultured; this process is repeated to fabricate a 3D structure. Using this process, researchers have succeeded in constructing 3D tissues having an EC network structure by improving the cell-coating steps. Because nanofilm coating technology can be used to fabricate 3D tissues layer by layer, it cannot increase the thickness dynamically. In cell sheet technology, the thickness of a 3D tissue can be increased in units of several numbers of cell layers. Cell sheet technology uses cell culture dishes coated with the thermoresponsive polymer poly(*N*-isopropylacrylamide) (PIPAAM) with nanometer-level thickness [30]. These dishes allow cultured cells to be detached from the culture surface as cell sheets at a temperature of less than 32 °C. Simple ordinal cell culture methods with temperature-responsive culture dishes can be used to engineer 2D cell sheets under adequate temperature conditions. Preserved adhesive factors in fabricated 2D cell sheets are advantageous for re-adhesion during layering through adhesive factors released by the cells using the gelatin-gel stamp technique [31]. The stamp techniques allow several number of cell sheets layering for an hour. Fabricated 3D tissues with layered cell sheets can also be manipulated by highly intelligent tools [32].

As described above, prompt blood flow can be achieved in engineering of 3D tissues with cell sheet technology after transplantation because of preserved EC network during the fabrication of 3D tissues [7]. The network can be established as immature vessels in transplanted 3D tissues within 24 h after transplantation. Even when only coculturing ECs and mesenchymal cells within Matrigel, at least 3 days are required to supply blood flow [33]. Thus, cell sheet technology can create dense 3D tissues with vascularization in vivo by exploiting the functions of the cells. In vitro EC networks and in vivo blood perfusion are achieved more quickly through cell sheet technology than scaffold or nanofilm coating technology (Table 1).

**Table 1:** Comparison of EC-network assembly technologies. This table indicates the comparison of the period of EC network formation and connection to host blood circulation after transplantation among three assembly technologies, scaffold, and nanofilm coating and cell sheet technology

Technology	EC	EC network cultivation periods	Cocultured cells	Ratio of EC	Function as blood vessels checked in vivo	References
Scaffold (PLLA-PLGA)	HUVEC	3~7 days	Fibroblast Skeletal muscle cells	10~80%	Done (day 10)	[9, 26]
Nano film coating	HUVEC	3 day	Fibroblast, MSC, iPS, myocardial cells	9%	non	[29]
Cell sheet	Rat EC, HUVEC,	1〜3 days	Fibroblast, SMC, myocardial cell	8~10%	Done (within 24 h)	[7, 8, 12]

Comparison of EC-network assembly technologies

Additionally, vessels within tissues align and organize naturally into appropriate shapes and structures in vivo. Patterning techniques have been actively studied to create 2D shapes by micropatterning cell adhesive areas or nonadhesive areas on the surfaces of cell culture materials [34]. Microprinting of adhesive protein on the surface has also been achieved with polydimethylsiloxane (PDMS) micropatterning technology [35]. For lining cells in a specific direction, culture dishes having microgroove grids have also been studied [36]. These 2D patterning techniques could be combined with cell sheet layering methods to create precise 3D structures. However, patterning at the micrometer or nanometer level, i.e., smaller than the size of a cell (less than approximately 10 μm), tends to make cells disorganized within 3D cell-dense tissues. Moreover, 2D patterning structures can be modified easily by the surrounding cells. Compared with micropatterning technology, 3D bioprinting of cell-shaping scale is larger than micropatterning. Although a delicate pattern cannot be created, techniques that can control the amount of blood vessels arranged in a 3D tissue are expected to be suitable for intentional blood vessel guidance into 3D tissues [37].

Native patterning and ECM could be used for tissue engineering with decellularized scaffold technique. Decellularized tissues are then recellularized with vascular ECs and perfused in vitro and in vivo. The kidneys of animals were decellularized and reseeded with human target cells [38]. Since the cell engraftment and infiltration of recellularization are affected by the decellularization protocol [39], further studies are needed to allow application of this technology.

These assembly methods have benefits and disadvantages (Table 2), and the appropriate method must be chosen based on the target tissue characteristics and applications. Because assembly technology will develop really day by day, we have to obtain information and arrange them adequately for target tissues.

**Table 2:** Assembly technology. The table shows several advantages and disadvantages of assembly technologies

Technology	Vascularization engineered 3D tissues	Advantage	Disadvantage	References
(1) 3D scaffold-based technology	Self-organization within scaffold or recellularization native vasculature ECM within decellularized tissues	Controlled selforganization with scaffold characters or native ECM and shape	With exogenous ECM or animal experiments	[38]
(2) Cell sheet technology	Self-organization within layered cell sheets	Without exogenous scaffolds	Specific manipulation	[31]
(3) Nanofilm coating technology	Self-organization within laded cells	Without specific equipment	Manipulation of 3D tissues to transplantation	[29]
(4) 3D printer technology	Vascular shaping with 3D printing	Fabrication free artificial shape	Patterning size limitation and degradation for long cultivation	[37]

### Perfusion for maturation of vasculature within 3D tissues: fabrication perfusable basement for perfusion stimulation within vasculature within 3D tissues

**Fig. 4:**
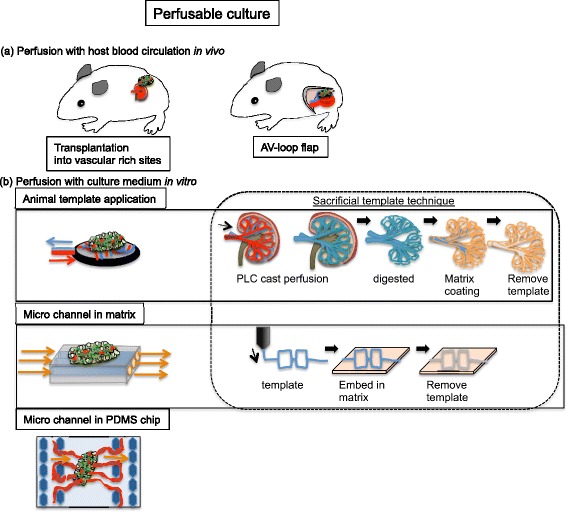
Perfusable culture technology. The figure illustrates representative perfusion culture technology for fabrication of 3D tissues with vasculature

Blood vessels function to transport blood throughout tissues and organs. During the embryonic stage, after vascularization, redundant vessels are remodeled [40]. Thus, if blood perfusion does not occur through vessel or EC networks, they should be removed as redundant vasculature. Researchers have used traditional approaches to perfuse 3D tissues, including transplantation into animals to exploit biological circulation. In the selection of transplantation position, highly vascular sites, e.g., the kidney capsule, are usually chosen. However, engineered tissues have to be re-transplanted for therapeutic application. Accordingly, in the field of plastic surgery, the arteriovenous (AV) loop has been used to make a flap for promotion of vascularized 3D fabricated tissues [41, 42], allowing retransplantation into another site for maturation of 3D tissues by vascular anastomosis. Recently, vascular beds made from rat femoral tissues were perfused ex vivo, and 3D myocardial tissue was developed using cell sheet technology [43] (Fig. 4a).

In recent studies, microchannels within biodegradable scaffolds or ECM gel, such as collagen or fibrin, have been fabricated for perfusion into the channel. These microchannels have been employed in “body-on-a-chip” technology with PDMS microprocessing [44]. In our laboratory, a collagen gel microperfusable basement was vascularized by cell sheet technology [45]. Furthermore, microperfusable tubes were endothelialized with cells derived from cell sheets. Compared with the natural circulation system, these perfusable gel structures have no paracrine effects. By cocultivation with MSC inner scaffolds or ECM gel, it becomes possible to establish an effective perfusable basement for 3D tissue containing blood vessels without using animals. Microchannel fabricated by 3D printer with water-soluble polymer (poly vinyl alcohol: PVA) was also used as sacrificed template technique with embedding gelatin gel. The channel scale was more than 1 mm [46]. Perfusable vasculature under 100-μm diameter was also microfabricated by EC encapsulation with polymer by hydrodynamic shaping and photopolymerization. After embedded matrix, the microfabricated vessel could make branches from them [47]. More natural complex vasculature were tried to fabricate with perfusion poly caprolactone (PLC) cast into natural kidney vasculature. They digested kidney tissues without PLC cast and coated with collagen matrix. Finally, the PLC cast was removed as sacrificed template and remained complex structure of hollow collagen scaffolds (Fig. 4b animal material-applicated sacrificial template). They could be used as perfusable microvasculature basement for engineered 3D tissues [48]. Perfusion stimulation causes biomechanics for maturation of vasculature within 3D tissues. However, perfusion medium has to be conditioned well. Especially, oxygen delivery carrier replaced to erythrocytes was important to maintain and maturate tissues [49]. Since these methodologies have also advantages and disadvantages (Table 3), we have to choose and combine these technologies according to suitable microenvironment for vascularization of target 3D tissues. In fact, vascularized cardiac cell tissues could be obtained in vivo and in vitro with perfusable cultivation [43, 45]. Moreover, 3D vascularized engineered tissues were reported to be obtained with perfusion culture for 2 weeks in vitro [50, 51]. Thus, multistep vascularized tissue engineering is one of actualizing strategies for fabrication of functional vascularized 3D tissues.

**Table 3:** Perfusion culture technology. The table shows several advantages and disadvantages of perfusion culture technologies

	Technique	Advantage	Disadvantage	References
(a) Host blood circulation	Transplantation into rich vasculature sites	Without high technique and prompt vascularization	The size of transplantation tissues have limitation	[7, 8]
	AV-loop flap	Prompt vascularization and retransplantation with vascular anastomosis	Necessity of technique for anastomosis	[41, 42]
(b) Perfusion culture medium	Animal template application	Native vasculature can apply	Difficulty of maintaining animal template for long time in vitro	[43]
	Microchannel in matrix and on chip	Animal-free experiments	Necessity of promotion vascularization ability	[45]

## Conclusion

In the fields of regenerative medicine and drug discovery, vascularized 3D tissues are needed for continued progress and the development of effective treatments. Key points for inducing vasculature within 3D tissues are selection of cells and materials, assembly methods, and perfusion techniques. In the past few decades, many technologies have been produced for generation of vascularized 3D tissues. Because there are numerous options for engineering 3D tissues, it is necessary to make an appropriate selection considering the specific target tissue. At the point to choose them, it is essential to understand suitable or native microenvironment for the target-tissue situation in vivo. The biomimetic microenvironment of target tissues will induce adequate cell-cell interaction, distance, cell morphology, and function within tissues. For fabrication of the microenvironment, multistep combination technologies might be a candidate of an actual strategy for vascularization within 3D tissues. It could progress for fabrication of vascularized 3D tissues and give us the generation functional tissues. We hope that these artificial tissue or organs will facilitate the development of effective treatment strategies for patients with intractable diseases in the future.

## Abbreviations

2D: Two-dimensional
3D: Three-dimensional
ADMSC: Adipose-derived MSC
AV: Arteriovenous
bFGF: Basic fibroblast growth factor
BMMSC: Bone marrow-derived MSC
EC: Endothelial cell
ECM: Extracellular matrix
EPC: Endothelial progenitor cell
HGF: Hepatocyte growth factor
HUVEC: Human umbilical vein endothelial cell
iPS: Induced pluripotent stem
MSC: Mesenchymal stem cell
PDGF-BB: Platelet-derived growth factor-BB
PDMS: Polydimethylsiloxane
PGA: Poly glycolic acid
PIPAAM: Poly(*N*-isopropylacrylamide)
PLA: Poly lactic acid
PLC: Poly caprolactone
PLGA: Poly(lactide-co-glycolide)
PLLA: Poly(L-lactide)
PVA: Poly vinyl alcohol
SMC: Smooth muscle cell
TE: Tissue engineering
VEGF: Vascular endothelial growth factor

## References

[1] Nishida K, Yamato M, Hayashida Y, Watanabe K, Yamamoto K, Adachi E, Nagai S, Kikuchi A, Maeda N, Watanabe H, Okano T, Tano Y (2004). Corneal reconstruction with tissue-engineered cell sheets composed of autologous oral mucosal epithelium. N Engl J Med.

[2] Sawa Y, Miyagawa S (2013). Cell sheet technology for heart failure. Curr Pharm Biotechnol.

[3] Colton CK (1995). Implantable biohybrid artificial organs. Cell Transplant.

[4] Bersini S, Yazdi IK, Talo G, Shin SR, Moretti M, Khademhosseini A (2016). Cell-microenvironment interactions and architectures in microvascular systems. Biotechnol Adv.

[5] Kim JJ, Hou L, Huang NF (2016). Vascularization of three-dimensional engineered tissues for regenerative medicine applications. Acta Biomater.

[6] Shimizu T, Sekine H, Yang J, Isoi Y, Yamato M, Kikuchi A, Kobayashi E, Okano T (2006). Polysurgery of cell sheet grafts overcomes diffusion limits to produce thick, vascularized myocardial tissues. FASEB J.

[7] Sekiya S, Shimizu T, Yamato M, Kikuchi A, Okano T (2006). Bioengineered cardiac cell sheet grafts have intrinsic angiogenic potential. Biochem Biophys Res Commun.

[8] Takeuchi R, Kuruma Y, Sekine H, Dobashi I, Yamato M, Umezu M, Shimizu T, Okano T (2016). *In vivo* vascularization of cell sheets provided better long-term tissue survival than injection of cell suspension. J Tissue Eng Regen Med.

[9] Levenberg S, Rouwkema J, Macdonald M, Garfein ES, Kohane DS, Darland DC, Marini R, van Blitterswijk CA, Mulligan RC, D'Amore PA, Langer R (2005). Engineering vascularized skeletal muscle tissue. Nat Biotechnol.

[10] Takebe T, Sekine K, Enomura M, Koike H, Kimura M, Ogaeri T, Zhang RR, Ueno Y, Zheng YW, Koike N, Aoyama S, Adachi Y, Taniguchi H (2013). Vascularized and functional human liver from an iPSC-derived organ bud transplant. Nature.

[11] Takasato M, Er PX, Chiu HS, Maier B, Baillie GJ, Ferguson C, Parton RG, Wolvetang EJ, Roost MS, Lopes SM, Little MH (2016). Kidney organoids from human iPS cells contain multiple lineages and model human nephrogenesis. Nature.

[12] Sekiya S, Muraoka M, Sasagawa T, Shimizu T, Yamato M, Okano T (2010). Three-dimensional cell-dense constructs containing endothelial cell-networks are an effective tool for in vivo and in vitro vascular biology research. Microvasc Res.

[13] Sasagawa T, Shimizu T, Sekiya S, Haraguchi Y, Yamato M, Sawa Y, Okano T (2010). Design of prevascularized three-dimensional cell-dense tissues using a cell sheet stacking manipulation technology. Biomaterials.

[14] Sasagawa T, Shimizu T, Sekiya S, Yamato M, Okano T (2014). Comparison of angiogenic potential between prevascular and non-prevascular layered adipose-derived stem cell-sheets in early post-transplanted period. J Biomed Mater Res A.

[15] Ikhapoh IA, Pelham CJ, Agrawal DK (2015). Atherogenic cytokines regulate VEGF-A-induced differentiation of bone marrow-derived mesenchymal stem cells into endothelial cells. Stem Cells Int.

[16] Caplan AI, Correa D (2011). The MSC: an injury drugstore. Cell Stem Cell.

[17] Roh JD, Sawh-Martinez R, Brennan MP, Jay SM, Devine L, Rao DA, Yi T, Mirensky TL, Nalbandian A, Udelsman B, Hibino N, Shinoka T, Saltzman WM, Snyder E, Kyriakides TR, Pober JS, Breuer CK (2010). Tissue-engineered vascular grafts transform into mature blood vessels via an inflammation-mediated process of vascular remodeling. Proc Natl Acad Sci U S A.

[18] Spiller KL, Anfang RR, Spiller KJ, Ng J, Nakazawa KR, Daulton JW, Vunjak-Novakovic G (2014). The role of macrophage phenotype in vascularization of tissue engineering scaffolds. Biomaterials.

[19] Auletta JJ, Eid SK, Wuttisarnwattana P, Silva I, Metheny L, Keller MD, Guardia-Wolff R, Liu C, Wang F, Bowen T, Lee Z, Solchaga LA, Ganguly S, Tyler M, Wilson DL, Cooke KR (2015). Human mesenchymal stromal cells attenuate graft-versus-host disease and maintain graft-versus-leukemia activity following experimental allogeneic bone marrow transplantation. Stem Cells.

[20] Nomi M, Atala A, Coppi PD, Soker S (2002). Principals of neovascularization for tissue engineering. Mol Asp Med.

[21] Chiu LL, Radisic M (2010). Scaffolds with covalently immobilized VEGF and Angiopoietin-1 for vascularization of engineered tissues. Biomaterials.

[22] Liu B, Li X, Liang G, Liu X (2011). VEGF expression in mesenchymal stem cells promotes bone formation of tissue-engineered bones. Mol Med Rep.

[23] Tayalia P, Mooney DJ (2009). Controlled growth factor delivery for tissue engineering. Adv Mater.

[24] Horikoshi-Ishihara H, Tobita M, Tajima S, Tanaka R, Oshita T, Tabata Y, Mizuno H (2016). Coadministration of adipose-derived stem cells and control-released basic fibroblast growth factor facilitates angiogenesis in a murine ischemic hind limb model. J Vasc Surg.

[25] Langer R, Vacanti JP (1993). Tissue engineering. Science.

[26] Lesman A, Koffler J, Atlas R, Blinder YJ, Kam Z, Levenberg S (2011). Engineering vessel-like networks within multicellular fibrin-based constructs. Biomaterials.

[27] Lesman A, Gepstein L, Levenberg S (2014). Cell tri-culture for cardiac vascularization. Methods Mol Biol.

[28] Kaushiva A, Turzhitsky VM, Darmoc M, Backman V, Ameer GA (2007). A biodegradable vascularizing membrane: a feasibility study. Acta Biomater.

[29] Amano Y, Nishiguchi A, Matsusaki M, Iseoka H, Miyagawa S, Sawa Y, Seo M, Yamaguchi T, Akashi M (2016). Development of vascularized iPSC derived 3D-cardiomyocyte tissues by filtration layer-by-layer technique and their application for pharmaceutical assays. Acta Biomater.

[30] Shimizu T (2014). Cell sheet-based tissue engineering for fabricating 3-dimensional heart tissues. Circ J.

[31] Haraguchi Y, Shimizu T, Sasagawa T, Sekine H, Sakaguchi K, Kikuchi T, Sekine W, Sekiya S, Yamato M, Umezu M, Okano T (2012). Fabrication of functional three-dimensional tissues by stacking cell sheets in vitro. Nat Protoc.

[32] Tadakuma K, Tanaka N, Haraguchi Y, Higashimori M, Kaneko M, Shimizu T, Yamato M, Okano T (2013). A device for the rapid transfer/transplantation of living cell sheets with the absence of cell damage. Biomaterials.

[33] Cheng G, Liao S, Kit Wong H, Lacorre DA, di Tomaso E, Au P, Fukumura D, Jain RK, Munn LL (2011). Engineered blood vessel networks connect to host vasculature via wrapping-and-tapping anastomosis. Blood.

[34] Tsuda Y, Shimizu T, Yamato M, Kikuchi A, Sasagawa T, Sekiya S, Kobayashi J, Chen G, Okano T (2007). Cellular control of tissue architectures using a three-dimensional tissue fabrication technique. Biomaterials.

[35] Tanaka N, Ota H, Fukumori K, Miyake J, Yamato M, Okano T (2014). Micro-patterned cell-sheets fabricated with stamping-force-controlled micro-contact printing. Biomaterials.

[36] Zhou X, Hu J, Li J, Shi J, Chen Y (2012). Patterning of two-level topographic cues for observation of competitive guidance of cell alignment. ACS Appl Mater Interfaces.

[37] Mir TA, Nakamura M (2017). Three-dimensional bioprinting: toward the era of manufacturing human organs as spare parts for healthcare and medicine. Tissue Eng Part B Rev.

[38] Song JJ, Guyette JP, Gilpin SE, Gonzalez G, Vacanti JP, Ott HC (2013). Regeneration and experimental orthotopic transplantation of a bioengineered kidney. Nat Med.

[39] Faulk DM, Carruthers CA, Warner HJ, Kramer CR, Reing JE, Zhang L, D'Amore A, Badylak SF (2014). The effect of detergents on the basement membrane complex of a biologic scaffold material. Acta Biomater.

[40] Lenard A, Daetwyler S, Betz C, Ellertsdottir E, Belting HG, Huisken J, Affolter M (2015). Endothelial cell self-fusion during vascular pruning. PLoS Biol.

[41] Nau C, Henrich D, Seebach C, Schroder K, Fitzsimmons SJ, Hankel S, Barker JH, Marzi I, Frank J (2016). Treatment of large bone defects with a vascularized periosteal flap in combination with biodegradable scaffold seeded with bone marrow-derived mononuclear cells: an experimental study in rats. Tissue Eng Part A.

[42] Arkudas A, Beier JP, Pryymachuk G, Hoereth T, Bleiziffer O, Polykandriotis E, Hess A, Gulle H, Horch RE, Kneser U (2010). Automatic quantitative micro-computed tomography evaluation of angiogenesis in an axially vascularized tissue-engineered bone construct. Tissue Eng Part C Methods.

[43] Sekine H, Shimizu T, Sakaguchi K, Dobashi I, Wada M, Yamato M, Kobayashi E, Umezu M, Okano T (2013). *In vitro* fabrication of functional three-dimensional tissues with perfusable blood vessels. Nat Commun.

[44] Zhang B, Montgomery M, Chamberlain MD, Ogawa S, Korolj A, Pahnke A, Wells LA, Masse S, Kim J, Reis L, Momen A, Nunes SS, Wheeler AR, Nanthakumar K, Keller G, Sefton MV, Radisic M (2016). Biodegradable scaffold with built-in vasculature for organ-on-a-chip engineering and direct surgical anastomosis. Nat Mater.

[45] Sakaguchi K, Shimizu T, Horaguchi S, Sekine H, Yamato M, Umezu M, Okano T (2013). *In vitro* engineering of vascularized tissue surrogates. Sci Rep.

[46] Li S, Liu YY, Liu LJ, Hu QX (2016). A versatile method for fabricating tissue engineering scaffolds with a three-dimensional channel for prevasculature networks. ACS Appl Mater Interfaces.

[47] DiVito KA, Daniele MA, Roberts SA, Ligler FS, Adams AA (2017). Microfabricated blood vessels undergo neoangiogenesis. Biomaterials.

[48] Huling J, Ko IK, Atala A, Yoo JJ (2016). Fabrication of biomimetic vascular scaffolds for 3D tissue constructs using vascular corrosion casts. Acta Biomater.

[49] Seekell RP, Lock AT, Peng Y, Cole AR, Perry DA, Kheir JN, Polizzotti BD (2016). Oxygen delivery using engineered microparticles. Proc Natl Acad Sci U S A.

[50] Nashimoto Y, Hayashi T, Kunita I, Nakamasu A, Torisawa YS, Nakayama M, Takigawa-Imamura H, Kotera H, Nishiyama K, Miura T, Yokokawa R (2017). Integrating perfusable vascular networks with a three-dimensional tissue in a microfluidic device. Integr Biol (Camb).

[51] Pagliari S, Tirella A, Ahluwalia A, Duim S, Goumans MJ, Aoyagi T, Forte G (2014). A multistep procedure to prepare pre-vascularized cardiac tissue constructs using adult stem cells, dynamic cell cultures, and porous scaffolds. Front Physiol.

